# Heart Rate and Heart Rate Variability of Amateur Show Jumping Horses Competing on Different Levels

**DOI:** 10.3390/ani11030693

**Published:** 2021-03-04

**Authors:** Csaba Szabó, Zsolt Vizesi, Anikó Vincze

**Affiliations:** 1 Department of Feed and Food Biotechnology, Faculty of Agricultural and Food Sciences and Environmental Management, University of Debrecen, Böszörményi út 138, 4032 Debrecen, Hungary; vizesizsolt@gmail.com; 2 Department of Hippology, Faculty of Agricultural and Environmental Sciences, Szent István University Kaposvár Campus, Guba Sándor 40, 7400 Kaposvár, Hungary; vincze.aniko@szie.hu

**Keywords:** horse, show jumping, heart rate, heart rate variability

## Abstract

**Simple Summary:**

The increase in the heart rate and various heart parameters play an important role in assessing the fitness of sport horses. The fitness of a horse, that particular horse’s competition routine, and the resulting lower stress affect various cardiac parameters. The aim of this study was to examine the effect of the phases of competition (warm-up, resting period, show jumping course riding, cool-down) and the difficulty of a course (100, 120, 130 cm) on the heart rate and selected heart rate variability parameters of show jumping horses. The heart rate was monitored with a “Polar Equine heart rate monitor” before, during, and after a show jumping course was completed. Neither analysis of the average heart rate nor that of the maximum heart rate were able to detect a statistically proven difference among competition levels. In contrast, according to heart rate variability measures, such as maximum RR intervals, SD1, RMSSD, pNN50, and %VLF picked up differences in workload level. It has been confirmed that lower-class show jumping (up to 120 cm height) is not a strenuous exercise for horses.

**Abstract:**

Heart rate is one of the gold standards used to assess the workload level and fitness of horses. However, when slight differences need to be detected, it is not sensitive enough. Therefore, the aim of this study was to test the effect of competition level and phase of exercise on the heart rate and heart rate variability parameters in show jumpers. Fourteen horses were examined competing on three different levels: 100 cm (*n* = 4), 120 cm (*n* = 6), and 130 cm (*n* = 4). The length of work (min); average and maximum heart rate; average, maximum and minimum RR intervals (ms); SD1 and SD2 (ms); RMSSD (ms) and pNN50 (%); VLF, LF, HF (%) were analyzed. The measurement was divided into four phases: warm-up, resting period, show jumping course riding, and cool-down. The level of the course had no significant effect on average and maximum heart rates throughout the entire exercise. The maximum RR interval, RMSSD, pNN50, SD1, and %VLF values were significantly different (*p* < 0.05) in horses competing at 100 cm height from those competing in the 120 cm group. The SD1 value was sensitive for the level of competition, while the SD2 parameter was sensitive for detecting exercise phases. In conclusion, heart rate variability parameters are more sensitive for detecting smaller differences in workload than heart rate alone in lower-level show jumpers.

## 1. Introduction

Fitness denotes the obtaining of a specific metabolic status—as a result of training—which makes equine athletes capable of achieving good results in sport. Exercise has beneficial effects on the heart. As a result of regular training, the weight of the heart can be increased, its pump functions can be improved, and small capillaries develop and open in the heart muscle, thus increasing the oxygen supply to the heart [1]. As a result of these changes, the heart pumps blood throughout the body with less contractions. Individuals adapted to exercise show a lower resting heart rate and cardiac hypertrophy [2]. The heart rate is closely related to oxygen uptake and energy expenditure during exercise [3]. A higher heart rate can be associated with higher lactate concentrations, more faults, a lower technical score and closer take-off distance [4]. Lactate concentration is one of the most valuable parameters used to calculate VL4 (speed which corresponds to a blood lactate of 4 mmol/L), which changes during training progress [5]. The most developed techniques used today in race training, such as infrared thermography, also correlate with lactate concentration [6]. Also, other parameters may be used in training monitoring, such as changes in PBMCs (peripheral blood mononuclear cell) proliferation and activity [7] or cytokines mRNA expression [8]. High-performing horses have a quicker recovery after an exercise session [9]. Recording both the number of heart beats and the time between two heart beats (RR interval), gives the possibility to perform variability analyses, which has many diverse applications, including human medicine [10]. Heart rate variability (HRV) measures proved to be powerful, i.e., in detecting diseases and assessing the risk factors of diseases, as well as in the classification of disease severity in humans [10]. In equines, other parameters—such as serum amyloid A, which is the main acute phase protein—has proven to be useful for detection of some subclinical pathological alterations that can affect training and competition performance [11]. The promising results with HRV parameters in human studies most likely initiated several research studies using horses in response to exercise [12,13,14,15,16]. RR intervals can be measured conventionally with electrocardiogram (ECG) machines intended for use in hospitals, but this equipment is cumbersome and cannot be used in field conditions, especially on competing horses. Since the availability of portable, precise HRV monitors [17], a number of investigations of HRV on horses have been performed in the time domain at rest and at exercise [12,13,14,15,16]. Only one study was found to examine cardiac parameters during competition, from saddling to unsaddling of show jumping horses [18]. However, in this study, the authors did not report the difficulty of the course (the height of the jumps) and only RMSSD (root mean square of successive RR interval differences) and SDRR (standard deviation of all the R-R intervals) values were determined. It has been proven that the effect of training should be monitored in a competition environment as well [19]. Assessment of the fitness status of a horse in field conditions is, consequently, crucial.

Therefore, the aim of this study was to examine the effect of the phases of competition (warm-up, resting period, show jumping course riding, cool-down) and the difficulty of a course (100, 120, 130 cm) on the heart rate and selected heart rate variability parameters of show jumping horses in field conditions.

## 2. Materials and Methods

### 2.1. Animals

The procedures complied with the European Code of Practice for the care and use of animals for scientific purposes (DL n. 116, 27/01/1992) were considered to be low risk and did not require approval by Hungarian authorities. In this study, we recorded data from 18 horses, but after data checking, four readings had to be excluded due to lost signals. Data were collected from three different show jumping classes (100 cm—*n* = 4; 120 cm—*n* = 6; 130 cm—*n* = 4) during the winter indoor show jumping season (from October to February) in Hungary. The experiments were performed at two different locations of the tournament: the Pannon Equestrian Academy at Szent István University’s Kaposvár Campus (Hungary) and the Mátai stud farm, located in the town of Hortobágy (Hungary). All competitions were judged against the clock without a jump off with a course length between 450–550 m and a required speed of 325–350 m/min. Our previous study [20] revealed that lower-level show jumping (up to 120 cm height) is not a strenuous exercise for horses. Therefore, no measurements were made in 110 cm height classes, but the 100 cm high class was included as a kind of control. The horses were randomly selected (with the approval of their owners and riders), and sex was not considered in the selection of animals to be tested.

### 2.2. HR and HRV Recordings

Measurements were recorded with a POLAR EQUINE SCIENCE RS800CX (Polar Ltd., New York, NY, USA) kit containing a heart rate belt, heart rate sensor and transmitter (T56H), and with a sport watch for storing the data. The belt was submerged in water and the hair in the position of the electrodes was moisturized, according to the factory recommendation, in order to ensure a better heart rate reading. The signal receiving watch was attached to the front of the saddle, in order to have a stable distance from the data transmitter [17], to prevent signal loss. Recordings were made from the time of saddling up of the horse (putting a saddle on a horse), continuously during riding, and up to the time when the horse was unsaddled (remove the saddle from the horse). Although the watch provides the possibility to split the exercise into laps (phases) by pressing a button, the riders usually wear gloves, and they are not able to push the button properly. Therefore, this method seemed impractical. Therefore, an operator observed the rider continuously, using a watch with synchronized time and recording all data. The operator recorded the exact time of mounting, of the end of the warm up, of the start and of the completion of the course ride, and of dismounting. The measurement was divided into four phases: warm-up (from mounting to end of warming up, including walking, trot, canter and interval warm up jumps); resting period (between the end of warming up and starting to ride the course, which was walking from the training arena to the competition arena, trotting and cantering before the jumping competition course); show jumping course riding; and cool-down (from finishing the course till dismounting, mainly some trot and walking). Data from the watch was transferred to a PC computer, and raw data were evaluated by Polar Pro Trainer 5 (Polar Ltd., New York, NY, USA) software. The following data were recorded for the entire exercise and for all the phases: length of work (min); average and maximum heart rate; average, maximum and minimum RR intervals (ms); SD1 (Poincaré plot standard deviation perpendicular the line of identity, ms) and SD2 (Poincaré plot standard deviation along the line of identity, ms); RMSSD (root mean square of successive RR interval differences) and pNN50 (percentage of successive RR intervals that differ by more than 50 ms, %); VLF, LF, HF (oscillations into very-low-frequency (0.0033–0.04 Hz), low-frequency (0.04–0.15 Hz), and high-frequency (0.15–0.4 Hz) bands, %) were analyzed.

### 2.3. Statistical Analyses

The experimental data were evaluated by GraphPad Prism 7.05 (GraphPad Software Inc., San Diego, CA, USA) statistical software package with two-way ANOVA. Interaction was not significant in the case of any parameter; therefore, results were presented as pooled. In the case of significant main effect, the differences between the group means were tested using the Tukey test.

## 3. Results

There was no significant difference between the entire length of the exercise of horses competing at 100, 120, or 130 cm (*p* > 0.05) (Table 1). It could be observed that the show jumping competition means about 50–60 min work for the horses. The average and maximum heart rate failed to detect any effect resulting from the competition level (*p* > 0.05). There was no significant difference between the average RR intervals (time between two heartbeats), the minimum RR intervals, SD2, LF and HF parameters for horses competing at 100, 120, and 130 cm high obstacles (*p* > 0.05). However, the maximum RR, SD1, RMSSD, and pNN50 values were significantly higher in the 100 cm group than in the 120 cm group (*p* < 0.05). Significantly lower VLF values were observed for horses competing at 100 cm, compared to the horses competing at 120 cm and 130 cm (*p* < 0.05).

Riders spent an average 40 min with warming up (Table 2). An average of 4 min was spent between warming up and starting to ride the course. The time spent on the course corresponded to the time allowed. The cool-down phase (trotting and walking after completion of the course) was about 11 min on average. Not surprisingly, the highest average and maximum heart rate were detected while riding the competition course. The maximum heart rate was lowest during the resting and cool-down phase. In contrast, the average heart rate did not show more significant differences among the phases. RR intervals and SD2 values responded to the different work intensities of the phases of the exercise, while other parameters did not show significant differences. Average RR values even detected significant differences between the resting and cool-down phases. While the maximum heart rate did not differentiate between warm-up and course riding phases, average and maximum RR and SD2 successfully distinguished between them. Our results show that HRV variables respond differently according to the level of exercise or phase of an exercise: average RR, minimum RR, and SD2 are more sensitive to the phases of the exercise, while SD1, RMSSD, and VLF detect exercise level differences. Maximum RR values respond to both traits, but HF and LF percentages did not show any significant differences.

## 4. Discussion

It is widely accepted that warming-up prior to exercise or competition is essential for optimal performance. In our study, the riders spent about 38 min warming up. Whitaker et al. [21] indicate that significant differences (*p* < 0.001) are apparent between the total time spent warming up and the levels of competition. Tranquille et al. [22] measured an average warm-up time of 18 min for riders competing on a 135–145 cm track, which did not differ for a particular rider among days; however, there were inter-rider differences (range = 12–27 min). Even though the importance of the warm-up is widely accepted among human athletes and equine trainers as a mandatory tool for limiting injury and optimizing performance [23,24], these results show that the length of the warm-up time cannot be clearly related to the difficulty of competition.

Becker-Birck et al. [18] obtained similar mean heart rate values compared to our results for show jumping horses during competition. Interestingly, the increased difficulty of the competition seems to decrease the maximum heart rate (Table 1), even if it was not significant in our case. Horses competing in equestrian disciplines, particularly in higher level show jumping, are required to have high technical skills, but higher heart rate values do not necessarily coincide with higher speeds [25,26], which may reflect heightened emotional reactivity as well [27]. Other researchers observed that well-conditioned show jumpers participating regularly in equestrian competitions were not stressed compared with less experienced horses [2,28] and all measured stress indicators (heartbeats, cortisol, glucose, and lactate concentrations in saliva) measured and recorded during an entire competitive season were higher in younger (less experienced) horses, than in older (more experienced) horses [29]. Oldruitenborg et al. [30] found that ponies with a lower maximum heart rate were less prone to injury. These results indicate that the heart rate and heart rate variability response not only depend on the strenuousness of the exercise, but also depend on the level of experience. Equestrian sports are about the cooperation between rider and horse. Therefore, there is a possibility that the rider influences the physiological responses in a horse to a given exercise. Matching horse-rider pairs had lower HR and behavioral scores [31], and jumping horses are tolerant to the level of experience of a rider [32] in terms of HR and HRV parameters. The weight of the rider can affect the behavioral, physiological, and gait parameters, but was not altered in a lower intensity dressage test [33]. These examples reveal that the effect of the rider on equine physiological responses needs further studies.

In our study, the average heart rate and maximum heart rate during the course ride were significantly higher than during the resting and cool-down phases. Similar heart rate trends have been previously observed by Lekeux et al. [34] in Belgian Saddlebreds competing in the Belgian Championship and by Bazanno et al. [25] in Italian Saddlebred mares competing in an outdoor national jumper class at a height of 135 cm. Furthermore, Bazanno et al. [25] found that the fence height during warming up causes a significant increase (*p* = 0.0158) in heart rate when shifting from 100 cm to 125 cm jumps. These results show an increased heart rate caused by an increase in physical activity when the horses have a similar level of experience.

Heart rate variability (HRV) is the physiological phenomenon of variation in the time interval between heartbeats, expressed as several parameters. One measure is the variation in the beat-to-beat interval (RR interval). The lowest RR interval and lowest values for SDRR (standard deviation of all the RR intervals), RMSSD, and SD1 were found when riders were mounting young horses [35]. At the same time, cortisol levels in saliva changed in the opposite way. During these times, the horses did not show physical activity, such as during lunging or when being ridden. The researchers concluded that the reduced RR intervals and other HRV parameters in horses associated with mounting by a rider indicate stress. Visser et al. [36] also found that stress in horses leads to a reduction in the variability of the RR interval. In our study, the maximum RR interval decreased significantly between the warm-up and the resting phase (1948 ms vs. 1001 ms) and was lowest (but not significantly) during the riding of the course (609 ms). This finding may indicate that horses were anticipating the competition, and a stress response occurred. Similarly, von Lewinski et al. [14] found different heart beats and RMSSD responses to the same dressage program during rehearsal and performance in public. This phenomenon happened even when both horses and riders were very well accustomed to the circumstances.

None of the SDRR and RMSSD traits differed significantly between horses competing in dressage and show jumping, while the heart rate was lower in dressage horses than in show jumpers at the end of the competition and immediately thereafter [18]. The difference in heart rate most likely reflects different physical demands. Furthermore, Becker-Birck et al. [18] found decreases in the RMSSD occurred only during the second and the third day of a three day show jumping competition, where the demands of the competition increased each day. In our study, we recorded RMSSD and pNN50 values, both show considerably decreased values at 120 cm competitions, compared to 100 cm high exercise. However, we measured higher values for horses competing at 130 cm level. As a result, the difference was not significant in the lowest class group of horses. Lower RMSSD values were strongly linked to more calmness, as proved by lavender aromatherapy [37]. Horses competing at a lower level of competition are generally younger (less experienced) or not such good equine athletes; therefore, the stress factor during the exercise is most likely stronger. This theory is supported by the results of Younes et al. [15], who found higher levels of RMSSD both at rest and during exercise in four-year-old endurance horses, compared to five- or six-year-old ones. In our case, it seems that the difficulty of the competition in higher show jumping classes may start to play a larger role. Therefore, it would be interesting to conduct a study with higher level (140–160 cm) show jumpers, as well. Interestingly, these parameters detect no statistically proven differences among the phases of the exercise. Frick et al. [16] detected a similar result with healthy, normally performing and poor performing horses on treadmill training, where RMSSD was sensitively reflect treatment, but not phase difference. 

Geometric variables SD1 and SD2 showed a differential response. Average SD1 values were more sensitive to the level of competition, while SD2 parameters showed changes with the phases of the exercise. Schmidt et al. [35] found that the changes in SD1 closely related to those in RMSSD, but in our case, this was only observed in relation to the overall exercise average values.

Significantly lower VLF proportions were observed for horses competing at 100 cm (%VLF 70.5) compared to horses competing at 120 (%VLF 86.2) and 130 cm (%VLF 87.5). Baldwin and Chea [37] observed the decrease in %VLF as a result of lavender aromatherapy, where lavender effectiveness was tested as a calming agent for horses. The %HF parameter is associated with calmness; the higher value means a more stress-free animal. Experimental results suggest that both LF and HF powers tended to be higher at night than during the day, when HRV is recorded over a 24-h period during stall rest [38]. Baldwin-Chea [34] also showed higher LF and HF values under the influence of lavender aromatherapy. Based on these results, we conclude that the 120 cm high course causes more stress to the horses than a 100 cm high course, while the additional difficulty of the course of 130 cm does not mean additional stress for the equine athletes. Quite interestingly, these parameters did not show any differences among the phases.

## 5. Conclusions

The average and maximum heart rates are not sensitive enough for workload differences between lower -level show jumping competitions to be detected. Our results show that heart rate variability parameters are differentially affected by the level of competition and by the phases of the exercise; therefore, these more sensitively detect differences. Heart rate variability parameters—especially %VLF—also confirm that the 100 cm jumping course is not a particularly strenuous exercise for show jumping horses. Nevertheless, we have to emphasize that these findings are related to amateur level riders and involved a small sample of horses. Therefore, studies with more and higher-level equine athletes are needed to fully explore the relationships among HR, HRV, exercise strenuousness, and other influencing factors.

## Figures and Tables

**Table 1:** The effect of the difficulty of the course on the heart rate and heart rate variability parameters of show jumper horses.

Parameters	Level of the Course (Difficulty)			RMSE *	*p*
	100 cm	120 cm	130 cm		
Length of exercise (min)	48.3	54.5	61.4	9.0	0.1665
Average heart rate (bps)	102.5	110.8	107.9	21.7	0.5000
Maximum heart rate (bps)	170.8	152.9	152.2	31.5	0.1624
Average R-R (ms)	672.3	609.6	682.1	155.9	0.2813
Minimum R-R (ms)	381.5	414.3	444.0	105.9	0.2585
Maximum R-R (ms)	1592 ^a^	1141 ^b^	1209 ^a,b^	538.9	0.0355
SD1 (ms)	69.2 ^a^	14.9 ^b^	18.6 ^a,b^	64.3	0.0283
SD2 (ms)	240.7	194.0	213.2	112.5	0.4443
RMSSD (ms)	97.9 ^a^	21.1 ^b^	26.3 ^a,b^	91.0	0.0284
pNN50 (%)	8.2 ^a^	1.9 ^b^	3.1 ^a,b^	7.5	0.0386
VLF (%)	70.5 ^a^	86.2 ^b^	87.5 ^b^	14.9	0.0027
LF (%)	16.6	10.9	10.2	7.7	0.0377
HF (%)	6.0	3.0	2.3	5.9	0.1689

^a,b^ Means with the same letter are not significantly different (*p* > 0.05); * RMSE route mean square error.

**Table 2:** The effect of phases of the exercise on the heart rate and heart rate variability parameters of show jumper horses.

Parameters	Phase of the Exercise				RMSE *	*p*
	Warm Up	Rest	Course Riding	Cool Down		
Length of section (min)	37.8 ^a^	4.2 ^c,d^	1.3 ^d^	11.4 ^b^	4.9	0.0001
Average heart rate (bps)	88.6 ^b^	95.3 ^b^	172.9 ^a^	73.6 ^b^	21.7	0.0001
Maximum heart rate (bps)	168.6 ^a,b^	143.7 ^b,c^	193.2 ^a^	126.0 ^c^	31.5	0.0001
Average R-R (ms)	695.9 ^a,b^	672.6 ^b^	390.9 ^c^	833.5 ^a^	155.9	0.0001
Minimum R-R (ms)	362.8 ^b^	434.1 ^a,b^	335.5 ^b^	521.2 ^a^	105.9	0.0002
Maximum R-R (ms)	1948 ^a^	1001 ^b^	609 ^b^	1600 ^a^	538.9	0.0001
SD1 (ms)	24.8	19.4	30.3	51.3	64.3	0.4662
SD2 (ms)	382.4 ^a^	176.0 ^b^	52.8 ^c^	240.3 ^b^	112.5	0.0001
RMSSD (ms)	35.1	27.4	42.9	72.7	91.0	0.4659
pNN50 (%)	2.9	3.0	3.6	6.6	7.5	0.4410
VLF (%)	80.4	86.2	80.5	81.2	14.9	0.6203
LF (%)	10.1	10.9	15.3	13.1	7.7	0.2056
HF (%)	3.1	2.9	4.3	4.4	5.9	0.8832

^a,b,c,d^ Means with the same letter are not significantly different (*p* > 0.05); * RMSE route mean square error.

## Data Availability

The data presented in this study are available on request from the corresponding author. The data are not publicly available due to institutional policy.
